# Supplementary material for: Understanding General Practitioners’ Antibiotic Prescribing Decisions in Out-of-Hours Primary Care: A Video-Elicitation Interview Study
Source: Antibiotics (Basel). 2020 Mar 7;9(3):115. doi: 10.3390/antibiotics9030115 (PMC7148451; doi:10.3390/antibiotics9030115)
Supplement: Supplementary file 1 [file antibiotics-09-00115-s001.zip › Supplementary material S2_topic guide_Elicitation interviews OOH.pdf]

Supplementary material 1: Elicitation interview

Part 1:

1/ Why did you choose this consultation?

2/ What went well?

3/ What could have gone better?

Part 2:

⇨ describe thought processes and feelings at key points

Before the video:

1/ Why did this patient come to you?

2/ What was his/her question?

During the video: stop at key points by the interviewer or the participant, (reflection on communication, thought process, etc.) max. a few minutes

1/ What are you doing at this point? Why (do you do/say what you do/say)?

2/ What are you feeling/thinking at this point? Why?

After the video:

1/ Did you meet the patients expectations? Why(not)? What did you feel/think?

2/ Why did you choose this action plan/treatment and when/why was your decision made during the consultation?

3/ What was your reasoning behind your treatment decision made during the consultation and what factors were taken into account?

Part 3:

⇨ Views and experiences of the consultation

1/ What did you find to be beneficial/challenging in the consultation ?

2/ What would you do differently? Why?

3/ Would this consultation be any different in your own practice (not out-of-hours)? Would your decision making be different?
