# Supplementary material for: Understanding General Practitioners’ Antibiotic Prescribing Decisions in Out-of-Hours Primary Care: A Video-Elicitation Interview Study
Source: Antibiotics (Basel). 2020 Mar 7;9(3):115. doi: 10.3390/antibiotics9030115 (PMC7148451; doi:10.3390/antibiotics9030115)
Supplement: Supplementary file 1 [file antibiotics-09-00115-s001.zip › Supplementary material table S1_ quotes _ elicitation interviews OOH_def.pdf]

Supplementary Material 1: Quotes

**Theme 1: Influence of unverified interpretation of information or assumptions**	
Exploring ideas, concerns and expectations	“I think in the beginning, and now we are going to talk really in communication terms, I didn’t align my ICE with his ICE enough, because he immediately started about the dyspnoea (patient had talked about some shortness of breath while biking), so I thought it’s more serious, but at the end his question was more he wanted to be examined thoroughly, and if it’s fine, it’s fine. And I thought from the beginning should I or shouldn’t I give antibiotics. And because of the fact he said at the end: Oh, that’s reassuring for me, I thought: ‘wow, luckily I don’t need to make the decision anymore, because he’s happy already, so I could have known that from the beginning.”<br>“I must say, it seems that objective criteria don’t determine my decision-making process, that’s really an eye-opener that I was not aware off”<br>(GP01, female, 25-30y)
	I: Can you describe what her question was, why she came?<br>P: Euhm, yes, I actually didn’t ask that specifically, I probably should have done that. I think she just, that her symptoms dragged on too long, and that she really thought… I want to get rid of them, and she really was in pain,… the cough,… really bothered her?” (GP18, female, 25-29y)
	“He said “I have had it last year as well, euhm, and I wanted to be sure that it was the same”. So maybe I should have asked why he was so afraid, do you worry about something?”<br>(GP02, female, 25-29y)
	“I guess he came because he wanted reassurance from as many people as possible. That’s wat I suppose. Because I couldn’t ask: did you not trust the judgement of your own doctor?” (Re-consultation) (GP16, male, 55-59y)
	P: “In my own practice I specifically ask about the ICE (ideas-concerns-expectations), are you worried or …? But know it was clear for me, I did not ask it, she came for the pain.”<br>I: “and you say, this is different at the GPC?”<br>P: “yes”<br>I: “Can you explain why this is different?”<br>P: “you are in a hurry, you want to be as efficient as possible,…”<br>(GP12, female, 25-29y)
	“It’s not that I don’t dare to ask it, but it makes the story a bit awkward, you don’t know the patient, otherwise (own practice) you would have had your medical file, and maybe there would have been a note, or that you would have known: “Oh he had chlamydia before.”, but if you don’t know the patient,… sometimes you can base yourself a bit on the looks, *laughs*, but I guess that’s not the way to detect Chlamydia is it?” (GP10, female, 25-29y)
	“I was thinking, does he has a GP? Why does he come here? But I didn’t say it out loud.”(GP14, male, 60-64y)
Patients’~~’s~~ presentation & Doctor~~’~~s’ interpretation	“If you look back, you could think if she just came in and said: doctor, I’m an air hostess, I have some vaginal irritation, I called my own GP yesterday, but it’s not completely over yet, it’s difficult for me to go to work, I need a certificate”… but now you examine her, you prescribe something new,… it wouldn’t have taken so long.” (GP08, male, 60-64y)
	“Maybe it was someone who went to the Tropics? Then there would have been a bigger a priori risk that there was something more dangerous going on. But she didn’t look like she was a Tropics traveller. … But in fact, if you don’t ask it, you actually don’t know.” (GP06, female, 35-39y)
	“I think at that point, because it was such a rational, cool, no drama, intelligent man, that has influenced my decision. If it was a drama queen I wouldn’t think about prescribing. So here it depended on the type of person, and the question he had.” (GP01, female, 25-30y)

	I: “You could have prescribed nothing. Was this an option for you?”<br>P: “yes, but, with this lady, I’m pretty sure she would come back the next day to the GPC.”<br>I: “Here (own practice) it is much easier, you know your patients, and you can say carry on as you’re already doing…. But she has been to her GP before, and in less than 15-16 hours she’s back and worried,…” (GP08, male, 60-64y)
	“But the child was not out of breath,… sometimes I guess we think a layperson knows more, …, it’s a sign that the mum was fed up with it, and she could not judge herself anymore about whether she had to let the child be checked out,..., bad night probably, but to come to the doctor on call? But maybe it counts ups, she wouldn’t’ have come for one child, but they are both sick,… and that her ability to cope,… was not big enough anymore at that time,…, so she stopped thinking and came in,…” (GP13, female, 35-39y)
	“She really looked as if she was in a lot of pain, she wasn’t being theatrically or anything, that just did not fit her. But I would have expect that she would have been much sicker,... or that she would have had much more fever.” (GP12, female, 25-29y)
	P: “Normally I don’t use the word antibiotics if I don’t have to, only if they have brought it up already.”<br>I: “O I think I missed that, did they said something already about wanting antibiotics?”<br>P: “yes, or maybe not the word antibiotics, but she said: doesn’t she needs anything stronger? Or something like that?” (GP13, female, 35-39y)
	“Maybe it was not really clear, if he expected antibiotics or not, for me it was easy it was clearly not bacterial,…, and I think he agreed, …, he wanted something to get better quicker, because he had to work the whole week.” (GP17, female, 35-39y)
**Theme 2: Dichotomous thinking and communication**	
	“Which pathology can present itself like this? It didn’t fit well in a viral or bacterial infection… I was thinking I don’t have a box to put this in.” (GP06, female, 35-39y)
	“When I look at myself I feel I interrupt him too much, I ask all these questions, I would let him speak more freely in my own practice…. I have a tendency of not having enough focus in a consultation and I learned from colleagues, that at the GPC you just treat the complaint,… (GP02, female, 25-29y)
	And I thought from the beginning should I or shouldn’t I give antibiotics.” (GP01, female, 25-30y)
	“Now, I’m sure, … maybe I’ve used it too soon in the beginning, “it won’t be bacterial”, to reassure her. But now I have examined her abdomen…. I use these terms deliberately, because people ask antibiotics when they have infections, so I say it’s viral, it won’t work, but with a bacterial infection it sometimes does. … I hate it when people ask for antibiotics and you as a GP think it’s not necessary… So yes I try to anticipate that already by saying deliberately it’s viral. So medically it’s not necessary…. It’s a powerful way to say objectively it’s not necessary.” (GP06, female, 35-39y)
	“Now I think it’s some general flu-like-illness. I don’t know exactly if this is correct, but I often try to see, if it’s just the throat, and it looks like that….well, I’m more prone to say it’s bacterial instead of like a bit more spread symptoms, than it’s more viral, something ‘flu’-ish.” (GP15, female, 25-29y)
	“I still have the idea, I’m at the GPC, I only want to do urgency medicine. So I was thinking, ..., there were no acute stuff, she only was a bit worried, I just try to exclude the acute stuff. … That’s really a difference with patients here (own practice) or there.” (GP08, male, 60-64y)
	“I’m just listening to the patient at this moment, oh he wants to see if it is a throat infections, and I thought well if he says he doesn’t has any fever, the chance is small that there is really something serious. That’s really the difference

	at the GPC,… sometimes it are consultations of only 5 minutes, it has to move forward there, ..., it's mostly to check: is this serious or not, do I have to do something? At my own practice you think long term,... so at my own practice you look more into what does this patient want, but at the GPC you will say more "I think this will be necessary or I think this is not necessary" (GP17, female, 35-39y)
	"I could have said: stomach-intestine infection, without going into detail, but I really didn't want to suggest it was something bacterial, because for me it was certainly not. So for me it's important than to call it viral to the patient. … Because than you can explain that the body will fix that by itself, you can't do anything, it will be fine with some time, we make sure you're comfortable, so people know ok, my body will heal itself I won't have to do much, so that's reassuring." (GP06, female, 35-39y)
	I: You call it "a viral infection". Can you tell me why?<br>P: I think that's more clear, I just explain a virus enters the body and gives that rash, ... because there are these public campaign and posters about 'viral infections, no antibiotics, and so on'. If you call it that, and maybe they have seen them, they know already antibiotics won't work." (GP11, male, 40-44y)
	"I really use the words viral and bacterial. That really a reason to say, that's why I don't give you an antibiotics. It's a virus, it won't work against them, a lot of people understand then. Because often they think I'm sick I need antibiotics, so it's a good way to explain why you don't give any." (GP02, female, 25-29y)
	I: "So after the clinical examination. What are you thinking?"<br>P: "It looked something viral, the way he presented himself, no fever, bit off a hoarse voice, it's a bit the season, a lot of people are sick at this time, so I concluded it will be something viral." (GP17, female, 35-39y)
	"Body pain, that tends to be on the viral side more." (GP19, female, 50-54y)
	"I always explain why I don't give antibiotics and then I say because of resistance. But I don't know patients always understand this. I think here it was ok. But actually I should have said: "I don't give antibiotics because there are no alarm symptoms", because no antibiotics because of resistance sounds so ... on a higher level." (GP02, female, 25-29y)
**Theme 3: Safety netting: strategies and difficulties**	
	"Sometimes you can take the risk by saying: maybe it's not necessary (antibiotics). You could say wait 2 -3 days, and see if the symptoms go away…. But here you think: is he going to find help quickly or is he going to wait for another week. If he goes back to Paris, and the doctor over there says: those Belgian doctors, why didn't they help you properly ?" (GP09, male, 55-59y)
	"I think it's important to explicitly talk about alarm symptoms, because you don't know these patients, and there is a bigger chance that something more serious is going on. But in my own practice the content of this is quite the same.<br>… But I think in your own practice you can wait a bit longer, if you know the patients you're better at reading them." (GP20, female, 45-49y)
	"It was a difficult consult because it was at the GPC with a patient you don't know. I normally would wait for the results, but here I felt I had to give some medication already. I was constantly going through my options, it was very exhausting."<br>"I was not sure to give the antibiotics already. Because I thought maybe he won't return to his GP, and then there is an STD circulating around. I don't want to be responsible for that." (GP10, female, 25-29y)
	"You can't say: phone me tomorrow. That's not very loyal to your colleagues whose patient it is. And you can't say call your GP either tomorrow, I couldn't evaluate that over the phone, and he didn't see the patient today so he can't compare. … And sending them back for a consultation. Than they have to go twice. It feels like you're a bad doctor than. … And you can't call them yourself, that list will be too long." (GP20, female, 45-49y)

	"And with the language, it's more difficult to explain, there is less nuance, and you don't know what they will remember and how they will handle that information." (GP20, female, 45-49y)
	"I don't give a delayed prescription too often, I rather have the patient re-consulting me, but that's difficult at the GPC…. because you want to re-evaluate them yourself." (GP18, female, 25-29y)
	"You can always use the umbrella technique and say, well I don't take the risk and prescribe antibiotics anyway, but the fact they said they have their own GP and they will be able to reach him the next day reassures me to wait." (GP19, female, 50-54y)
	"You couldn't explain it well to the parents, and because it was so red, I thought it would be better if their own GP took another look. Just to be sure. And I didn't want to explain it in detail too much, because of the translation, and maybe then mum got scared." (GP20, female, 45-49y)
	"The fact that he does not have a GP, *sigh*, I would be more reassured I could safely sent him to one if he had any alarm symptoms. And then I wouldn't had to think so hard on whether I should give him a delayed antibiotic prescription or not." (GP02, female, 25-29y)
	"If it were people of my own practice, more highly educated, than I would explain more,… and I would have said: look I'm worried about a mastoiditis, and we can't' miss that. And that I'm worried, and that we will try to treat it ourselves, but if it should get worse they would have to see the ENT specialist. But now with the language issues, I thought, well, they understand the main things, the big picture, she knows she has to go to the emergency department, so I won't start with difficult terms." (GP12, female, 25-29y)
	"I still feel some uncertainty with the mother, and me as well… So I still have the feeling I need to repeat things." (GP21, male, 35-39y)

ICE: ideas concerns, expectations, GP: general practitioner, GPC: general practitioners cooperative
